# Pathology of lymph node tuberculosis in Yaounde: diagnostic agreement based on the Kappa coefficient

**DOI:** 10.11604/pamj.2018.30.158.14982

**Published:** 2018-06-22

**Authors:** Joseph Marie Mendimi Nkodo, Roger Ateba, Christiane Judith Ngo Pambe, Angèle Clarisse Kabeyene Okono, Jean Louis Essamé Oyono

**Affiliations:** 1 Department of Morphological Sciences and Anatomy Pathological, Faculty of Medicine and Biomedical Sciences of the University of Yaounde 1, Cameroon

**Keywords:** Tuberculosis, lymph nodes, histopathological technique, diagnostic agreement

## Abstract

**Introduction:**

Lymph node tuberculosis remains widespread in Cameroon. Our goal was to compare the diagnostic agreement between the hematein-eosin coloration technique and the Ziehl-Neelsen technique.

**Methods:**

This study is a retrospective and comparative study realized in the Yaounde University Teaching Hospital over a period of 5 years. We needed to specify the diagnostic agreement for tuberculosis, first inter observer and secondly between the standard and special colorations. The data we collected allowed us to determine the agreement rates observed and the kappa (k) coefficients with linear weighting.

**Results:**

The 186 samples of the 1726 cases of tuberculosis of all locations represented a proportion of 10.78%. There were more male patients (65.05%) with a sex ratio (M:F) of 1.30. The average age was 24.21 ± 15.5 with the extremes from 5 to 68 years. The most represented age group was from 10 to 39 years. The two observers agreed in 93 cases using hematein eosin coloration (P_0_ = 83.87 %; k = 0.8109) and on 73 samples using the Ziehl-Neelsen coloration (P_0_ = 89.78 %; k = 0.7734). The two coloration techniques presented an agreement on 104 samples (P_0_ = 88.17 %; k = 0.8783).

**Conclusion:**

The routine choice of the hematein eosin coloration technique not paired with the coloration technique of Ziehl-Neelsen can effectively alleviate the program for fighting tuberculosis in an environment of limited resources.

## Introduction

Tuberculosis (TB) is one of the 10 foremost causes of death in the world [1]. More than 95% of the deaths due to tuberculosis take place in African and Asian countries with low or intermediate income [2]. In Cameroon the national program for the fight against tuberculosis (PNLT) counted 26 110 cases of TB, all forms taken together, of which 15 080 were smear positive cases. This corresponds to a notification rate of 124 and 73 for 100 000 inhabitants respectively [3]. The lymph nodes outcome represents the most involved extra pulmonary locations in the countries with a high prevalence [4-6]. Thus, in front of all granulomatous lesions, it is the histopathology that permits to exclude all similar pathologies while identifying the causative agent after a special coloration [7]. Therefore the interest of the present study whose principal goal was to compare the diagnostic agreement between the standard coloration technique with the special Ziehl-Neelsen technique.

## Methods

**Figure 1:**
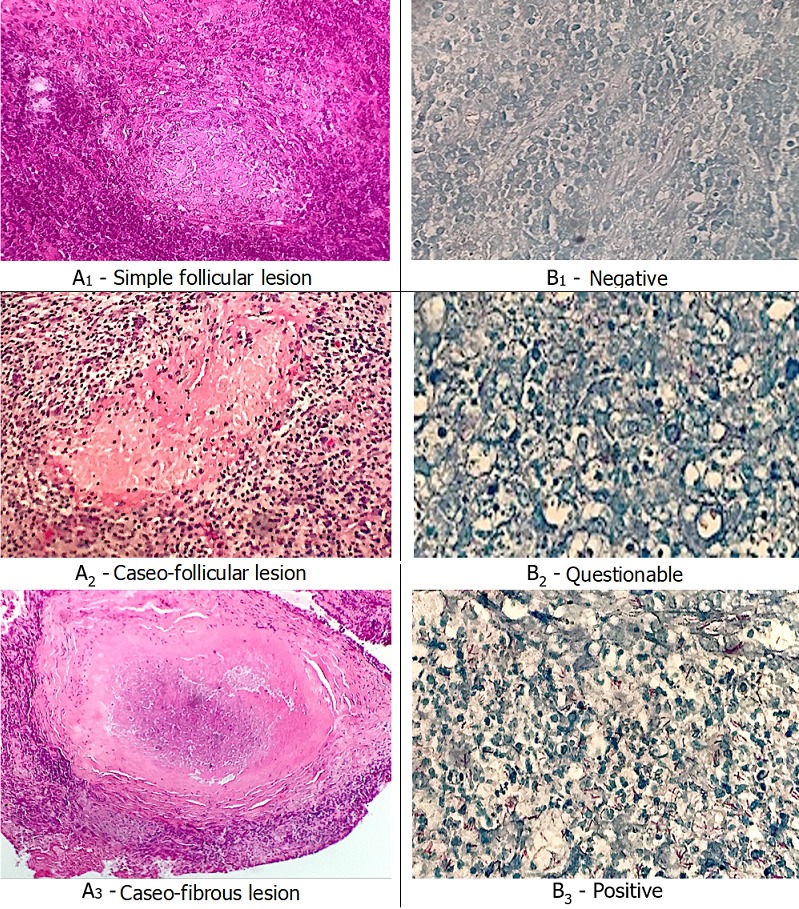
Microphotography: A 1-3, appearances of granuloma. Hematein eosin staining Gx250 (A1 and A2), Gx100 (A3); B 1-3, research of acid-alcohol resistant bacilli. Ziehl-Neelsen staining, Gx400 (B1 and B2), Gx250 (B3)

It was a retrospective and comparative study, realized in the anatomo-pathological laboratory of the Yaounde University Teaching Hospital. It covered a period of five years from January 1^st^, 2007 to December 31^st^, 2012. It included all lymph nodes samples for which tested positive in the tuberculosis diagnosis during the testing of histological sections after a hematein eosin staining. Also, paraffin blocks archived from these samples was supposed to be recorded and of good quality. All others lymph nodes samples which did not meet these conditions was excluded. We exploited archives of the histopathological reports in search not only for the diagnosis of lymph nodes tuberculosis or granulomatous adenitis, but also for socio-demographic data of patients from whom these lymph nodes samples had been taken. Archived slides only stained with hematein eosin were reexamined. The study executed two follow ups of the corresponding blocks and colored stained slides, one with hematein eosin and the other according to the Ziehl-Neelsen technique all in search of acid fast bacilli. This allowed to precise the diagnosis of tuberculosis. All the histological preparations however, obtained through this approach were analyzed by two pathologists with strict compliance to the double blind methodology. The goal was, first of all, to determine the agreement inter observers about the lesional diagnosis of three subgroups in terms of the appearance of the observed granulomas using standard coloration (Figure 1). Secondly, the study aimed specifying the diagnostic agreement of tuberculosis in each of the lymph node specimen corresponding to those different subgroups, first inter observer and then between standard and special coloration. To do so we needed to codify the responses of special coloration into three modalities structured as follows (Figure 1). The data obtained from the analysis and interpretation of all these preparations was reported in possibility tables for the purpose of determining the observed agreement rates and the kappa (k) coefficients with linear weighting. This data, determined with the observed agreements in mind and randomized, quantified the real agreement level between the inter observer results, firstly in terms of each coloration technique and secondly inter technique. The obtained values were then qualified as seen on Table 1.

**Table 1:** The qualifications of Kappa coefficient values

Agreement	Kappa Coefficients
Excellent	≥ 0,80
Satisfactory	0,60 ≤ κ < 0,80
Middle	0,40 ≤ κ < 0,60
Weak	0,20 ≤ κ < 0,40
Very weak	0 ≤ κ < 0,20
Great disagreement	< 0

## Results

**Figure 2:**
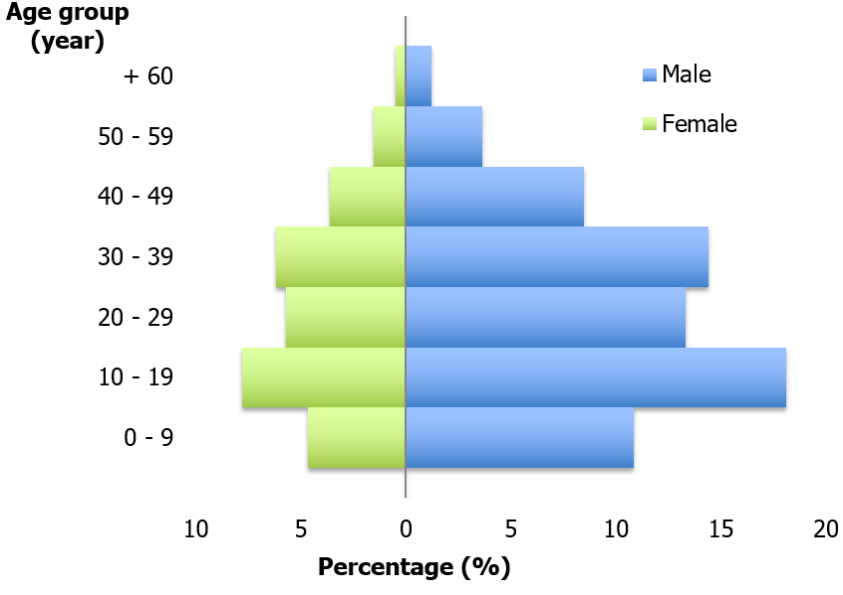
Distribution in the population in terms of age and sex

The exploitation of the archives of the Anatomo-pathological laboratory of our place of study allowed us to list 186 cases fulfilling our selection criteria, of a total of 1726 cases of tuberculosis in all locations together; this being a rate of 10.78%. The distribution of our sample according to sex showed that 121 samples came from male patients (65.05%) and 65 from female patients (34.95%), a sex ratio of 1.30 (**Figure 2**). The average age was 24.21 ± 15.5 with the extremes from 5 years to 68 years of age. We observed that the most represented age group was from 10 to 39 years of age (**Figure 2**). Of 186 kept cases two series of histological preparations corresponding to the coloration methods were examined. The quality of those colorations, as much for the hematein eosin as the Ziehl-Neelsen technique, was judged to be satisfactory by two observers. We recorded predominance in the cervical location of sampled lymph nodes at 86%; and 7% were found at axillary and inguinal locations. For the cases of cervical locations the patients of male sex were represented the most, with a rate of 46.5% against 39.6% of female patients. The axillary and inguinal locations had an even distribution between the sexes at 8.8 and 5.1% respectively. The results of the different classifications, which were meant to determine the agreement between the two observers on one hand and those of the two coloration types on the other, are presented in Table 2, Table 3 and Table 4. Using the hematein eosin coloration the observers agreed in 93 cases. The agreement rate P_0_ observed was 83.87%; for a confidence interval at 95% between 0.4262 and 0.5738. The maximum possible kappa with linear weighting, considering observed marginal frequencies, was 0.8109; for a confidence interval at 95% between 0.1167 and 0.3457. Using the Ziehl-Neelsen coloration technique they agreed in 73 cases. The agreement rate P_0_ observed was 89.78%; for a confidence interval at 95% between 0.3226 and 0.4669. The maximum possible kappa with linear weighting, considering observed marginal frequencies, was 0.7734; for a confidence interval at 95% between 0.0807 and 0.2903. The two coloration techniques presented a concordance in 104 cases. The agreement rate P_0_ observed was 88.17%; for a confidence interval at 95% between 0.4846 and 0.6312. The maximum possible kappa with linear weighting, considering observed marginal frequencies, was 0.8783; for a confidence interval at 95% between 0.2429 and 0.4739.

**Table 2:** The classification of the aspects of granuloma by the two observers

**Observer n°2**	**Observer n°1**				
	**Diagnostic**	1	2	3	**Total**
	1	43	10	11	64
	2	23	35	24	82
	3	18	7	15	40
	**Total**	84	52	50	186

**Table 3:** The classification of the presence of the acid-alcohol resistant bacilli by the two observers

**Observer n°2**	**Observer n°1**				
	**Diagnostic**	0	1	2	**Total**
	0	48	15	10	73
	1	33	11	13	57
	2	10	32	14	56
	**Total**	91	58	37	186

**Table 4:** The classification of the diagnosis by coloration or staining techniques

**Ziehl-Neelsen**	**Hemateine-eosin**				
	**Diagnostic**	1	2	3	**Total**
	0	52	19	20	91
	1	10	7	3	20
	2	14	16	45	75
	**Total**	76	42	68	186

## Discussion

If we take into account the number of laboratories of pathological anatomy which are functional in the city of Yaounde, the number of lymph node tuberculosis compiled over five years at the Yaounde University Teaching Hospital turned out to be very elevated (10.78%). This rate gives us a reliable statistical value for our study. The pleuro-pulmonary tuberculosis remains very frequent, but the occurrence of extra pulmonary forms is in constant rise as a result of the coinfection with the human immunodeficiency virus (HIV) [3]. These forms often show clinical and biological appearances which pose real diagnostic problems to the practitioners [7,8]. Due to this fact the lymph node tuberculosis deserves to be paid special attention in tropical regions. In clinical plans lymphadenopathies do not have etiological specificity and the clinician often has difficulties in separating benign and malignant lesions, especially lymphomatous ones [9]. Nevertheless, in the context of acute or chronic inflammations, the pathologist is confronted with a wide range of diagnostic methods. The main differential diagnoses for isolated adenitis are very diversified and include lymphoproliferative syndromes, infection with non-tubercular micro bacteria, fungal infections, bacterial adenitis, Kaposi's Sarcoma and carcinoma metastases [9]. Although the diagnosis basically rests on the morphological lesions discovered through standard coloration, the highlighting of bacteriological proof after special coloration remains very difficult as a result of the low level of bacilli in lymph node tuberculosis [10,11]. This fact might be due to the bad oxygenation of the lymph nodes as well as due to the importance of cell-mediated defence mechanisms at this level. Nonetheless, the evaluation of the delicacy of special coloration (the Ziehl-Neelsen technique), being very expensive and sometimes routinely required, turns out to be necessary in order to appreciate its usefulness in our working conditions [12]. In our samples tuberculous lymph nodes mainly occurs in male subjects with 65.05% of the cases, against 34.95% for female patients (Figure 1). This rate is contrary to that of 75% of women found by Chadir et al in Pakistan [13] and Béogo et al in Burkina Faso [14]; the difference is probably due to the inclusion criteria in their retrospective studies. They considered all cases of extra pulmonary tuberculosis whereas we only considered cases of tuberculous adenitis. The male predominance might, on the other side, also have been found by Elmghari et al [15] in Casablanca and Ngama et al in Lubumbashi [16]; and this in spite of different sampling sizes and the already addressed issues. The average age was 24.21 ± 15.5, with the extremes ranging from 5 years to 68 years of age. We observed that the most represented age groups were from 10 to 19 and then from 30 to 39 years of age (Figure 1). These high frequencies have also been described by various authors for children [15] and for adults [14,16]. This result, classically found in literature, shows that tuberculosis constitutes a drag to development because it affects the active population.

The quality of the samples is a precondition to that of the colorations, as much for the hematein eosin as the Ziehl-Neelsen technique, being judged to be satisfactory by two observers during our study. This is an essential condition for a diagnostic certainty, while otherwise the challenges remain numerous in countries of low income [7]. With respect to the topography, cervical lymph nodes were the most frequent in our series (86%). This tendency was also reported by various other authors [5,17,18]. This could be explained by the anatomical position and physiological role of the jugular-carotid lymph node chain being more accessible compared to the respiratory tract. The kappa coefficient is frequently used to test the reliability inter observers [19,20]. The importance of this reliability rests in the fact that it represents the measure in which the collected data in this study are correct and exact representations of the measured variables. For standard coloration the method used for measuring the reliability inter observers was the agreement rate, calculated as the number of agreed scores divided by the total number of scores. It was 83.87%. This allowed us to obtain a kappa with linear weighting of 0.8109; for a confidence interval at 95% between 0.1167 and 0.3457. This value shows an excellent agreement rate between two observers. The reliability of the evaluation of the presence of acid-fast bacilli in histological samples using Ziehl-Neelsen coloration for the two observers was also based on this detemination method, the agreement rate. Here it was 89.78%; for a confidence interval at 95% between 0.3226 and 0.4669. The kappa with linear weighting was 0.7734; for a confidence interval at 95% between 0.0807 and 0.2903. This value shows a satisfactory agreement between the two observers. Here the usage of the kappa coefficient should allow us to estimate the agreement or concordance between three diagnostic methods for the presence of acid-fast bacilli, driven by standard coloration on one side and the Ziehl-Neelsen coloration on the other side, in the absence of a precise reference. This in turn should allow the study of the reproducibility of this diagnostic method [19,21]. Thus, the observed agreement rate was 88.17%; for a confidence interval at 95% between 0.4846 and 0.6312. The kappa with linear weighting was 0.8783; for a confidence interval at 95% between 0.2429 and 0.4739. This value shows an excellent agreement rate between the two coloration techniques. The diagnostic agreement of lymph node tuberculosis shows precise and reproducible kappa coefficients while being variable. This overall good agreement rate can be compared to that found by Culter et al [10]. Nonetheless, during the interpretation of these values one should pay attention to the quality of the samples.

## Conclusion

During these last years remarkable progress was made in the histopathological diagnosis of tuberculosis. During these last years remarkable progress was made in the histopathological diagnosis of tuberculosis. Nevertheless, the diagnostic challenges of extra pulmonary tuberculosis in general and those of lymph node localization in particular still have to be mastered. The diagnosis of lymph node tuberculosis remains difficult due to the nature of the often-low level of bacilli infection during this sickness. If the hematein eosin coloration technique is readily realized in routine examinations in the majority of laboratories, the place to be assigned to the Ziehl-Neelsen technique, which is very expensive but affordable, entirely remains in environments with limited resources. Because the two techniques show good diagnostic agreement, the choice could also be about the standard coloration as a reference test in our working conditions. The choice could also effectively help the program for the fight against tuberculosis in its daily routine. This in turn would significantly contribute to improve the detection of lymph node tuberculosis cases and thus reduce the morbity and mortality of this disease in our countries.

### What is known about this topic

- Ganglionic tuberculosis remains a public health problem in Cameroon;
- The anatomo-pathological examination following a special and standard coloration remains an unavoidable diagnostic aid.

### What this study adds

- The evaluation of the diagnostic concordance of ganglionic tuberculosis through the Kappa Coefficient demonstrates a precision and inter-observers reproducibility and an inter-technique of staining that laboratories could exploit in cases of resource limited areas;
- This will permit to emit results which will always be reliable and will contribute to effectively relief the program for the fight against tuberculosis in routine screening.

## Competing interests

The authors declare no competing interest.
